# Cell surface antigen expression by peripheral blood monocytes in allergic asthma: results of 2.5 years therapy with inhaled beclomethasone dipropionate

**DOI:** 10.1155/S096293519600052X

**Published:** 1996-10

**Authors:** W. A. T. Slieker, P. Th. W. van Hal, J. M. Wijkhuijs, J. P. M. Hopstaken-Broos, J. A. Noordhoek, S. E. Overbeek, P. G. H. Mulder, D. S. Postma, H. C. Hoogsteden

**Affiliations:** 1 Department of Immunology Erasmus University Rotterdam and University Hospital Rotterdam-Dijkzigt P.O. Box 1738 Rotterdam 3000 DR The Netherlands; 2 Department of pulmonary Medicine Erasmus University Rotterdam and University Hospital Rotterdam-Dijkzigt P.O. Box 1738 Rotterdam 3000 DR The Netherlands; 3 Epidemiology and Biostatistics Erasmus University Rotterdam and University Hospital Rotterdam-Dijkzigt P.O. Box 1738 Rotterdam 3000 DR The Netherlands; 4 the Department of Pulmonary Medicine University Hospital Groningen The Netherlands

## Abstract

At present, inhaled glucocorticoids are widely accepted as the therapy of choice in chronic asthma. Treatment with inhaled glucocorticoids significantly suppresses local airway inflammation in asthmatics, but may also have systemic effects, e.g. a reduction of the number of circulating hypodense eosinophils or a down-modulation of HLA-DR antigen (Ag) expression by T lymphocytes in peripheral blood. However, the effect of long-term therapy with inhaled glucocorticoids on peripheral blood monocytes (PBM), which are the precursors of the most numerous cell type in the lung, the alveolar macrophage, have not yet been evaluated. We therefore investigated the expression of various cell surface Ag on PBM from non-smoking patients with allergic asthma who were treated for 2.5 years with a β_2_-receptor agonist plus either an inhaled glucocorticoid (beclomethasone dipropionate, BDP) (*n* = 4) or an anticholinergic or placebo (*n* = 8). We compared the results with healthy volunteers (*n* = 7). Long-term treatment of allergic asthmatics with inhaled BDP, but not anticholinergic or placebo therapy, was associated with a significantly lower CDllb Ag expression (*p* < 0.04) and higher expression of CD13, CD14 and CD18 Ag (*p* < 0.05, *p* < 0.02 and *p* < 0.04, respectively) when compared with the healthy control subjects (*n* = 7). Most interestingly, PBM of asthmatics treated with inhaled BDP expressed an almost two-fold higher level of CD14 Ag on their cell surface than PBM of patients treated with anticholinergic or placebo (*p* < 0.03). No significant differences in the expression of CD16, CD23, CD25, CD32 and CD64 Ag or HLA-DR were observed between PBM from the different patient groups or healthy controls. Taken together, this study shows that long-term local therapy with inhaled BDP coincides with an altered expression of at least one cell surface Ag on PBM from allergic asthmatics.


Mediators of Inflammation, 5, 362–369 (1996)

### Cell surface antigen expression by peripheral blood monocytes in allergic asthma: results of 2.5 years therapy with inhaled beclomethasone dipropionate

W. A. T. Slieker,[1,2,CA] P. Th. W. van Hal,[1,2] J. M. Wijkhuijs,[1] J. P. M. Hopstaken-Broos,[1] J. A. Noordhoek,[4] S. E. Overbeek,[2] P. G. H. Mulder,[3] D. S. Postma[4] and H. C. Hoogsteden[2]

Departments of [1]Immunology, [2]Pulmonary Medicine, [3]Epidemiology and Biostatistics, Erasmus University Rotterdam and University Hospital Rotterdam-Dijkzigt, P.O. Box 1738, 3000 DR Rotterdam, The Netherlands and the [4]Department of Pulmonary Medicine, University Hospital Groningen, The Netherlands

[CA]Corresponding Author
Tel: (+31) 10 408 8087
Fax: (+31) 10 436 7601

**At present, inhaled glucocorticoids are widely accepted as the therapy of choice in chronic asthma. Treatment with inhaled glucocorticoids significantly suppresses local airway inflammation in asthmatics, but may also have systemic effects, e.g. a reduction of the number of circulating hypodense eosinophils or a down-modulation of HLA-DR antigen (Ag) expression by T lymphocytes in peripheral blood. However, the effect of long-term therapy with inhaled glucocorticoids on peripheral blood monocytes (PBM), which are the precursors of the most numerous cell type in the lung, the alveolar macrophage, have not yet been evaluated. We therefore investigated the expression of various cell surface Ag on PBM from non-smoking patients with allergic asthma who were treated for 2.5 years with a $\beta_2$-receptor agonist plus either an inhaled glucocorticoid (beclomethasone dipropionate, BDP) ($n = 4$) or an anticholinergic or placebo ($n = 8$). We compared the results with healthy volunteers ($n = 7$). Long-term treatment of allergic asthmatics with inhaled BDP, but not anticholinergic or placebo therapy, was associated with a significantly lower CD11b Ag expression ($p < 0.04$) and higher expression of CD13, CD14 and CD18 Ag ($p < 0.05$, $p < 0.02$ and $p < 0.04$, respectively) when compared with the healthy control subjects ($n = 7$). Most interestingly, PBM of asthmatics treated with inhaled BDP expressed an almost two-fold higher level of CD14 Ag on their cell surface than PBM of patients treated with anticholinergic or placebo ($p < 0.03$). No significant differences in the expression of CD16, CD23, CD25, CD32 and CD64 Ag or HLA-DR were observed between PBM from the different patient groups or healthy controls. Taken together, this study shows that long-term local therapy with inhaled BDP coincides with an altered expression of at least one cell surface Ag on PBM from allergic asthmatics.**

**Key words:** Allergic asthma, Inhaled glucocorticoids, Peripheral blood monocytes

### Introduction

One of the major histopathological findings in asthma is chronic inflammation of the airways. In bronchial mucosal biopsies, this inflammation is characterized by an accumulation of mononuclear phagocytes, eosinophils, mast cells and T lymphocytes.[1–4] These inflammatory cells have also been described in the bronchoalveolar lavage fluid,[5–7] which, in addition, has been shown to contain elevated levels of various inflammatory mediators.[7,8] It is generally believed that the airway inflammation in asthma results from the concerted actions of the different types of inflammatory cells and their products, and underlies some of the clinical symptoms.[1,3,5]

The most numerous cell type in both the normal and asthmatic lung is the alveolar macrophage. This cell type is nowadays known to play a central role in initiating, perpetuating, and reducing inflammatory processes.[9–12] Monocytes and macrophages recovered from bronchoalveolar lavage fluid of asthmatics are highly activated,[13–15] manifested in the release of a large variety of mediators which in turn modulate the inflammatory responses of eosinophils, mast cells and T cells.[7,16] Furthermore, it has been reported that alveolar macrophages of asthmatic patients express elevated levels of

© 1996 Rapid Science Publishers low affinity receptors for IgE (CD23),[17] which are capable of inducing cellular activation in response to specific allergen.

At present, glucocorticoids are the most effective therapy for controlling airway inflammation and clinical symptoms in chronic asthma.[18,19] The precise mechanisms by which glucocorticoids reduce airway inflammation are not yet fully understood, but it is known that they modulate gene expression by binding to specific glucocorticoid-responsive elements in DNA.[20,21] One of the major mechanisms by which glucocorticoids inhibit inflammation is direct suppression of cytokine production by mononuclear phagocytes and T cells[22] or the induction of anti-inflammatory proteins such as lipocortin-1.[23] Additionally, glucocorticoids inhibit the production of pro-inflammatory mediators indirectly by suppressing the activity of several enzymes involved in the production of these mediators.

Therapy with inhaled glucocorticoids for 1–4 months results in a significant decrease in the numbers of macrophages, eosinophils, mast cells, and T cells in the bronchial epithelium and submucosa of asthmatic patients.[24–27] Only mild systemic effects have been reported with a daily dose of 1 000–2 000 μg of inhaled glucocorticoids. Numbers of peripheral blood eosinophils have been shown to be reduced after treatment with inhaled glucocorticoids.[19,28] The effect of long-term treatment with inhaled glucocorticoids on peripheral blood monocytes (PBM), the precursors of alveolar macrophages, has not yet been evaluated. Identification of the effects of therapy with inhaled glucocorticoids on peripheral blood cells may be important, as increased numbers of eosinophils and activated T cells are detected in blood of asthmatic patients, in addition to the infiltration of inflammatory cells in the airways.[29,30]

In this report, we analysed the expression of various cell surface Ag by PBM of non-smoking patients with allergic asthma who were treated for 2.5 years with an inhaled $\beta_2$-receptor agonist plus either inhaled beclomethasone dipropionate (BDP), or an anticholinergic or placebo. The patients were participants in the Dutch double-blind placebo-controlled CNSLD study.[31,32] Our data demonstrate that PBM of asthmatic patients treated with inhaled BDP, in contrast to PBM of patients treated with anticholinergic or placebo, show significant changes in the expression of CD11b, CD13, CD14 and CD18 Ag when compared with healthy control subjects. Importantly, PBM of asthmatics treated with inhaled BDP expressed significantly more CD14 Ag on their cell surface than PBM of patients who were treated with bronchodilator or placebo. These findings suggest that local inhaled glucocorticoid therapy, in addition to earlier reported effects on blood eosinophils,[19,28] may result in systemic effects on PBM.

### Materials and Methods

### Subjects

Twelve non-smoking allergic asthmatic patients (participating in the Dutch double-blind placebo-controlled CNSLD study)[31,32] and seven healthy volunteers were studied. The characteristics of each patient are described in Table 1. The diagnosis of asthma was made according to the criteria of the American Thoracic Society[33] and was based on a history of attacks of breathlessness and wheezing without chronic cough

**Table 1.** Patient characteristics

| Patient number | Gender | Age (years) | Baseline $FEV_1$ (% of predicted) | After 2.5 years $FEV_1$ (% of predicted) | Baseline $PC_{20}$ (mg/ml) | After 2.5 years $PC_{20}$ (mg/ml) |
|---|---|---|---|---|---|---|
| Glucocorticoid group | | | | | | |
| 1 | M | 36 | 46.3 | 64.5 | 0.13 | 1.46 |
| 2 | F | 44 | 75.0 | 88.5 | 0.06 | 0.45 |
| 3 | F | 45 | 49.2 | 69.6 | 0.05 | 0.21 |
| 4 | F | 60 | 59.7 | 90.3 | 0.06 | 0.96 |
| Median | | 44.5 | 54.5 | 79.1 | 0.06 | 0.71 |
| Anticholinergic/placebo group | | | | | | |
| 1 | F | 41 | 54.3 | 53.1 | 0.03 | 0.04 |
| 2 | M | 42 | 48.7 | 56.5 | 0.24 | 0.87 |
| 3 | M | 24 | 61.5 | 54.7 | 0.19 | 0.58 |
| 4 | M | 44 | 63.5 | 66.3 | 0.02 | 0.05 |
| 5 | M | 50 | 65.1 | 62.7 | 0.28 | 0.18 |
| 6 | M | 57 | 81.6 | 42.3 | 0.14 | 0.04 |
| 7 | F | 38 | 57.0 | 43.6 | 0.13 | 0.06 |
| 8 | M | 26 | 90.0 | 61.5 | 0.13 | 0.02 |
| Median | | 41.5 | 62.5 | 55.6 | 0.14 | 0.06 |

or sputum production. Chronic was defined as more than three months per year. At entry to the study, all patients showed a 20% decrease in $FEV_1$ resulting from inhalation of a provocative concentration of histamine of $\leq 8$ mg/ml ($PC_{20}$).[32,34] Allergy was defined as at least two positive wheal and flare reactions to skin prick tests with twelve common aeroallergens, or a positive test to house dust mite.[32] All patients but one had a baseline reversibility $\geq 10\%$ of predicted (one patient had a reversibility of 4%). Control subjects (three male, median age 27 years; four female, median age 23 years) were healthy volunteers, taking no medication, who had no history of allergy or asthma and had negative skin prick tests. Approval for the study protocol was obtained from the Medical Ethics Committees of the participating centres. All subjects gave written informed consent.

### Study design

Details of the study design have been described previously.[32] Briefly, after a double-blind randomization patients were treated with the $\beta_2$-receptor agonist terbutaline (two puffs of 250 µg q.i.d.) plus either: (A) inhaled glucocorticoid BDP, two puffs of 100 µg q.i.d., (B) anticholinergic bronchodilator (ipratropium bromide), two puffs of 20 µg q.i.d., or (C) placebo q.i.d. At the end of the study, no significant differences with regard to $FEV_1$ and $PC_{20}$ were found between the groups receiving either ipratropium bromide or placebo. Therefore, the data of these groups were pooled for analysis as one single group (designated as anticholinergic/placebo group). Heparinized venous blood of patients was collected after completing 2.5 years of treatment. Heparinized blood of healthy volunteers served as control.

### Isolation of peripheral blood mononuclear cells

Mononuclear cells from heparinized venous blood were isolated by Ficoll density centrifugation (Ficoll Paque; density 1.077 g/ml; Pharmacia, Uppsala, Sweden) for 15 min at room temperature (centrifugal force $1\,000 \times g$). Peripheral blood mononuclear cells (PBMC) were washed twice at 4°C using PBS (300 mosmol; pH 7.8) supplemented with 0.5% heat-inactivated BSA (Organon Teknika; Turnhout, Belgium) and 0.05% w/v $NaN_3$ (PBS/BSA/$NaN_3$). The cell concentration was adjusted to $5 \times 10^6$ cells/ml.

### Immunofluorescence staining

MoAb used in this study are listed in Table 2. CD11 and CD18 moAb were used because of the central role of leukocyte adhesion molecules in the recruitment of blood cells to inflammatory sites.[35] Cell surface aminopeptidase-N can be recognized by CD13 moAb, and may play a role in the inactivation of some

**Table 2.** Monoclonal antibodies used in this study

| moAb | Clone (isotype) | Source |
|---|---|---|
| CD3 | Leu-4 ($IgG_1$) | BD[a] |
| CD4 | Leu-3a ($IgG_1$) | BD |
| CD8 | Leu-2a ($IgG_1$) | BD |
| CD11b | 44 ($IgG_1$) | Dr N. Hogg[b] |
| CD13 | Q20 ($IgG_{2a}$) | Dr C. E. van der Schoot[c] |
| CD14 | UCHM1 ($IgG_{2a}$) | Dr N. Hogg |
| CD15 | VIM-D5 (IgM) | Dr W. Knapp[d] |
| CD16 | Leu-11b (IgM) | BD |
| CD18 | LFA-1/1 ($IgG_1$) | CLB, Amsterdam, the Netherlands |
| CD20 | B1 ($IgG_{2a}$) | Coulter Clone, Hialeah, FL |
| CD23 | Tü1 ($IgG_1$) | Biotest, Dreieick, Germany |
| CD25 | IL-2R ($IgG_1$) | BD |
| CD32 | IV.3 ($IgG_{2b}$) | Medarex Inc., West Lebanon, NH |
| CD64 | 32.2 ($IgG_1$) | Medarex Inc. |
| HLA-DR | L243 ($IgG_{2a}$) | BD |
| Anti-macrophage Ag | RFD9 ($IgG_1$) | Dr L. W. Poulter[e] |
| Isotype control | ($IgG_1$) | BD |
| Isotype control | ($IgG_{2a}$) | BD |
| CD1[f] | Leu-6 ($IgG_{2b}$) | BD |

[a]Becton Dickinson, Sunnyvale, CA, USA.
[b]London, UK.
[c]CLB, Amsterdam, the Netherlands.
[d]Vienna, Austria.
[e]Royal Free Hospital School of Medicine, London, UK.
[f]Used as $IgG_{2b}$ isotype control.

inflammatory mediators.[36,37] CD14 moAb was used because it recognizes the receptor for LPS binding protein, which may determine the cellular responsiveness to bacterial products.[38] moAb recognizing Fc$\gamma$ receptors (CD16, CD32, CD64) were used because these receptors play a role in the response of cells to specific antigens.[39] The presence of low affinity receptors for IgE, recognized by CD23 moAb, may lead to activation of cells in response to specific allergens.[40] Both the expression of the $\alpha$-chain of the IL-2 receptor (CD25) and HLA-DR are markers of cellular activation.[27] RFD9 recognizes a cell membrane determinant present on alveolar macrophages, but virtually absent on blood monocytes, and may be used as a marker of maturation.[41] Irrelevant isotype-matched antibodies were used as controls. Reagents were diluted to optimal concentration in PBS/BSA/$NaN_3$. All incubations (30 min each) were carried out on ice. Each incubation was followed by two washes with PBS/BSA/$NaN_3$ at 4°C. For single-colour stainings, aliquots of $2.5 \times 10^5$ PBMC (in a volume of 50 μl) were first incubated with 50 μl moAb, washed, and subsequently incubated with FITC-conjugated goat anti-mouse Ig (GAM-FITC; CLB, Amsterdam, the Netherlands). After the last washing procedure, the cells were resuspended in PBS/BSA/$NaN_3$. For two-colour stainings, 50 μl PBMC were incubated with both a FITC-conjugated and PE-conjugated moAb, washed and eventually resuspended in FACSFLOW™ (Becton Dickinson, Sunnyvale, CA, USA).

### Flow cytometry

Flow cytometric analyses were performed using a FACScan flow cytometer (Becton Dickinson) equipped with a 488 nm argon laser. Residual erythrocytes, dead cells and debris were excluded from analysis by electronic gating on the basis of forward and perpendicular light scatter. At least 7 500 events were acquired. Cell surface fluorescence was analysed after setting scatter gates on either the monocyte or lymphocyte fraction. Within the monocyte gate, cell surface fluorescence was expressed as molecules equivalent to soluble FITC (MESF). MESF values ($\times 10^4$) were obtained by interpolating cell surface fluorescence to a standard curve prepared using microspheres of known fluorescence intensities (Flow Cytometry Standards, Research Triangle Park, NC, USA). MESF values were corrected for background fluorescence by subtracting the MESF values obtained with isotype-matched control antibodies. Within the lymphocyte gate, results were expressed as the percentage of positively stained cells relative to an isotype-matched control antibody. Subpopulations of T lymphocytes were identified by two-colour labelling.

### Statistical analysis

Differences between groups in either the percentage of positively stained lymphocytes or the level of cell surface fluorescence by monocytes were compared using the two-tailed Mann–Whitney *U*-test. Differences associated with $p$ values $< 0.05$ were regarded as statistically significant.

### Results

### Long-term treatment with inhaled BDP in asthma is associated with differences in the level of cell surface Ag expression by PBM

The expression of various cell surface Ag by PBM from both non-smoking allergic asthmatics treated for 2.5 years with either inhaled BDP or anticholinergic/placebo, and healthy volunteers is summarized in Table 3. PBM from patients who received anticholinergic/placebo did not show any significant alterations in the levels of cell surface Ag expression when compared with PBM from healthy controls. In contrast, PBM from patients treated with inhaled BDP differed significantly in the level of expression of CD11b ($p < 0.04$), CD13 ($p < 0.05$), CD14 ($p < 0.02$), and CD18 ($p < 0.04$) Ag when compared with PBM from healthy controls (Fig. 1). A reduced level of CD11b Ag expression was observed, whereas the expression of CD13, CD14, and CD18 Ag was higher. The only significant difference between the two groups of asthmatic patients concerned the level of CD14 Ag expression; PBM from patients who had received inhaled BDP expressed significantly higher levels of CD14 Ag than PBM from asthmatic patients who had received anticholinergic/placebo (Table 3, Fig. 1). Both groups of patients and the control group, however, did not differ in the relative numbers of $CD14^+$ monocytes in peripheral blood (data not shown). The expression of RDF9 by PBM of asthmatics treated with inhaled BDP was significantly increased when compared with PBM of healthy controls ($p < 0.01$). However, this specific cell surface fluorescence fell within two-fold the fluorescence intensity of an isotype-matched control moAb, suggesting that monocytes express negligible levels of RFD9 Ag. No differences in the expression of CD16 (Fc$\gamma$RIII), CD23 (Fc$\varepsilon$RII),

**Table 3.** Specific cell surface fluorescence by peripheral blood monocytes

| | Control group ($n=7$) | Anticholinergic/placebo group ($n=8$) | Glucocorticoid group ($n=4$) |
|---|---|---|---|
| PBMC | | | |
| % CD15+ | 0.7 (0.5–2.0)[a] | 1.8 (0.4–5.5) | 1.6 (0.5–6.8) |
| Monocytes | | | |
| CD11b | 19.2 (9.6–27.1)[a,b] | 12.6 (2.1–26.4) | 7.6 (4.1–13.0)[c($p<0.04$)] |
| CD13 | 5.3 (1.5–6.4) | 5.4 (3.6–12.9) | 8.2 (6.1–18.7)[c($p<0.05$)] |
| CD14 | 13.0 (9.4–18.7) | 11.9 (7.9–19.7) | 20.4 (13.7–21.5)[c($p<0.02$)d($p<0.03$)] |
| CD16 | 1.1 (0.6–2.3) | 0.9 (0.6–1.6) | 1.3 (0.7–1.6) |
| CD18 | 8.5 (5.7–11.0) | 13.8 (6.3–33.4) | 12.5 (8.8–16.1)[c($p<0.04$)] |
| CD23 | 1.0 (0.9–1.6) | 1.1 (0.5–2.2) | 1.3 (0.7–1.8) |
| CD25 | 0.9 (0.7–1.3) | 0.9 (0.5–1.3) | 1.1 (0.6–1.6) |
| CD32 | 11.7 (6.6–15.0) | 13.4 (7.2–19.9) | 14.0 (11.5–17.6) |
| CD64 | 7.7 (4.3–10.4) | 8.4 (4.2–12.4) | 7.0 (6.3–10.8) |
| RFD9 | 1.3 (0.9–1.4) | 1.4 (0.6–2.4) | 1.8 (1.7–2.0)[c($p<0.01$)] |
| HLA-DR | 5.9 (3.8–6.6) | 6.8 (4.8–21.4) | 7.0 (5.7–9.3) |

[a]Median values (range).
[b]MESF values, corrected for background fluorescence by isotype-matched control Ab, were calculated using a calibration line obtained with microspheres of known fluorescence intensities. Statistical analysis was performed using the two-tailed Mann–Whitney *U*-test.
[c]Significantly different from control group.
[d]Significantly different from anticholinergic/placebo group.

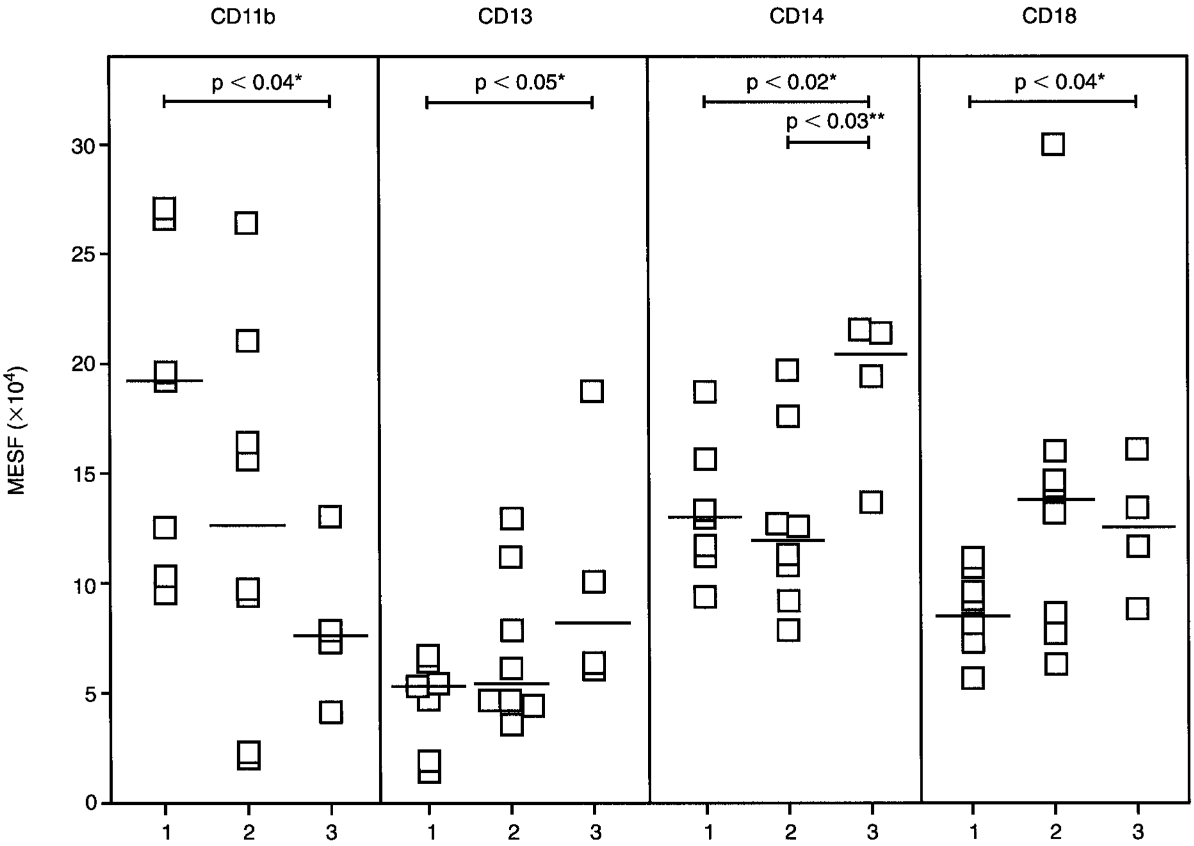

FIG. 1. Expression of CD11b, CD13, CD14 and CD18 cell surface Ag (expressed as MESF $\times 10^4$) by PBM. Median values are represented as a horizontal dash (—). *Value of glucocorticoid group (3) differs significantly from control group (1). **Value of glucocorticoid group (3) differs significantly from anticholinergic/placebo group (2).

CD25 (IL-2R), CD32 (FcγRII) and CD64 (FcγRI) were observed between both groups of patients and healthy controls. The expression of CD16, CD23, and CD25 Ag fell within two-fold the background fluorescence of isotype-matched control moAb, comparable with the expression of RFD9 Ag.

### Long-term therapy with inhaled BDP and differences in subsets of peripheral blood lymphocytes

In addition to analysing the cell surface Ag expression on PBM, we investigated whether long-term therapy with inhaled BDP is associated with differences in the percentage of T and B lymphocytes in peripheral blood (Table 4). No differences were observed in the percentages of either $CD4^+$ or $CD8^+$ T cells. However, both groups of patients had higher percentages of HLA-$DR^+$ T cells in their peripheral blood than healthy controls. Treatment with inhaled BDP did not alter the percentage of these activated T cells. Interestingly, patients who received anticholinergic/placebo, but not the patients treated with inhaled BDP, had a significantly higher percentage of B lymphocytes ($CD20^+$ cells) in their peripheral blood compared with healthy controls (Table 4).

### Discussion

In this study, we show an almost two-fold higher expression of CD14 Ag on PBM from allergic asthmatics treated for 2.5 years with an inhaled glucocorticoid (BDP, 800 μg daily) compared with patients treated with anticholinergic or placebo. In addition, long-term treatment of asthmatic patients with an inhaled glucocorticoid, but not anticholinergic or placebo, was associated with a significantly reduced expression of CD11b Ag on PBM, and a significantly increased expression of CD13, CD14, and CD18 Ag compared with healthy control subjects.

Glucocorticoids were introduced in 1949 as a new and promising drug in the treatment of inflammatory diseases.[42] Shortly thereafter, local administration of glucocorticoids was commenced to by-pass the unwanted systemic side effects.[43] Nowadays, inhaled glucocorticoids are widely accepted as the treatment of choice in chronic asthma.[28] Several reports showed that inhaled glucocorticoids are safe and almost free of systemic effects. However, high doses of inhaled glucocorticoids may still influence the hypothalamus-pituitary-adrenal axis.[28,44] More evidence for systemic effects of inhaled glucocorticoids comes from studies on circulating eosinophils and T cells. Circulating hypodense eosinophils, which have been shown to exhibit a great inflammatory potential, were reduced in asthmatic subjects after short-term treatment with inhaled glucocorticoids.[45,46] Furthermore, a small but significant reduction in HLA-DR expression by peripheral blood T cells has been reported after 6 weeks of therapy with inhaled glucocorticoids.[27] To our knowledge, modulation of PBM by inhaled glucocorticoids has not been described up till now.

Leukocyte adhesion molecules, among which the CD11/CD18 family of $\beta_2$ integrins, play an important role in the recruitment of cells from peripheral blood to the site of inflammation as well as in other immunological and inflammatory processes that require direct cellular interactions.[35,47,48] Both the expression and function of adhesion molecules are augmented in response to inflammatory mediators and cytokines.[49] The reduction in CD11b Ag expression by PBM observed here in asthmatic subjects treated with inhaled glucocorticoid, may have indirectly resulted from the local glucocorticoid-mediated reduction in inflammatory mediators in the lung. Alternatively, systemically absorbed BDP may be directly responsible for the modulation of CD11b Ag expression. In contrast to a reduced expression of CD11b, a slightly but significantly elevated CD18 Ag expression was seen on PBM of asthmatics treated with inhaled glucocorticoid when compared with PBM of healthy control subjects. The CD18 Ag is found on the cell surface in association with one of three different α chains, i.e. the CD11a, CD11b, or CD11c Ag.[47] Since the expression of CD11a and CD11c Ag were not analysed in this study, we do not know whether the observed increase in CD18 Ag expression coincides with an increased expression of CD11a and/or CD11c Ag.

Long-term treatment of asthmatics with inhaled BDP was associated with an increased expression of CD13 Ag by PBM compared with healthy controls. CD13 Ag, a cell membrane-bound aminopeptidase-N, has been shown to play an important role in modulating the activity of regulatory oligopeptides.[36] An in-

**Table 4.** Lymphocyte subpopulations in peripheral blood

| | Control group ($n=7$) | Anticholinergic/placebo group ($n=8$) | Glucocorticoid group ($n=4$) |
|---|---|---|---|
| Lymphocytes | | | |
| % $CD20^+$ | 4.2 (3.1–9.1)[a] | 7.2 (3.5–10.1)[b] ($p<0.04$) | 7.6 (3.9–16.4) |
| % $CD3^+$ | 67.4 (57.1–82.2) | 68.3 (52.6–81.9) | 73.5 (53.4–79.0) |
| % $CD3^+/CD4^+$ | 34.5 (22.0–44.1) | 40.6 (29.1–53.9) | 38.0 (32.1–40.9) |
| % $CD3^+/CD8^+$ | 23.8 (16.3–30.2) | 21.2 (11.3–35.7) | 33.0 (13.9–42.7) |
| % $CD3^+$/HLA-$DR^+$ | 1.0 (0.4–2.1) | 2.0 (1.2–9.3)[b] ($p<0.03$) | 5.6 (2.0–8.8)[b] ($p<0.02$) |
| Ratio CD4/CD8 | 1.6 (0.7–2.1) | 1.7 (0.8–3.6) | 1.2 (0.8–2.6) |

[a]Median values (range).
[b]Significantly different from control group.

crease in CD13 Ag expression may therefore enhance the cell's capacity to inactivate harmful inflammatory peptides. In this context, it is noteworthy that CD13 Ag expression by purified PBM increases upon *in vitro* culture in the presence of IL-4, suggesting that this cytokine, like glucocorticoids, has potential anti-inflammatory effects.[37,50] Future studies are needed to determine whether the increased expression of CD13 Ag observed here is accompanied by an increased aminopeptidase-N activity.

The CD14 Ag expression by PBM of patients treated with inhaled glucocorticoid was significantly increased compared with PBM of patients who received anticholinergic/placebo and healthy control subjects. It has been shown that CD14 Ag can function as a receptor for LPS.[38] In addition, a role for CD14 Ag has been implicated in the adhesion of monocytes to cytokine-activated endothelial cells.[51] CD14 Ag expression decreases upon differentiation/ maturation of monocytes into alveolar macrophages.[38] This process can be inhibited by glucocorticoids.[41,52] Therefore, it may be that in this study the interference of inhaled BDP with the maturation of PBM manifests itself in the increase of CD14 Ag expression.

In this study, we also show that all asthmatic patients, treated with either inhaled glucocorticoid or anticholinergic/placebo, have significantly increased percentages of HLA-DR$^+$ T cells in their peripheral blood when compared with healthy control subjects. Long-term treatment with inhaled BDP did not reduce the percentage of circulating activated (HLA-DR$^+$) T lymphocytes in both groups of patients. Recently, Wilson *et al.*[27] showed in an uncontrolled study that patients treated for 6 weeks with inhaled BDP showed a reduction in the percentage of activated T cells. Compared with our study, they used a higher dose of BDP and they did not compare their result with patients who did not receive inhaled glucocorticoids. In the present study, we also observed that patients treated with bronchodilator/placebo, but not the patients treated with inhaled BDP, had a higher percentage of B lymphocytes in peripheral blood compared with healthy controls. Therefore, treatment with inhaled glucocorticoid may have influenced the proportion of circulating B lymphocytes in our group of patients.

In summary, our data demonstrate that long-term therapy with an inhaled glucocorticoid coincides with an altered expression of at least one cell surface Ag on PBM of allergic asthmatics. In the treatment of patients clinicians should be aware of these systemic effects of inhaled glucocorticoids. Future studies are needed to determine whether changes in cell surface Ag expression by PBM are part of the anti-inflammatory action of inhaled glucocorticoids, and whether they correlate with the glucocorticoid-induced improvement in $FEV_1$, $PC_{20}$ and clinical symptoms.

### References

1. Jeffery PK, Wardlaw AJ, Nelson FC, Collins JV, Kay AB. Bronchial biopsies in asthma. An ultrastructural, quantitative study and correlation with hyperreactivity. *Am Rev Respir Dis* 1989; **140**: 1745–1753.
2. Djukanovic R, Roche WR, Wilson JW, *et al.* Mucosal inflammation in asthma. *Am Rev Respir Dis* 1990; **142**: 434–457.
3. Bradley BL, Azzawi M, Jacobson M, *et al.* Eosinophils, T-lymphocytes, mast cells, neutrophils and macrophages in bronchial biopsies from atopic asthma: comparison with non-asthma and normal controls and relationship to bronchial hyperresponsiveness. *J Allergy Clin Immunol* 1991; **88**: 661–674.
4. Poston RN, Chanez P, Lacoste JY, Litchfield, Lee TH, Bousquet J. Immunohistochemical characterization of the cellular infiltration in asthmatic bronchi. *Am Rev Respir Dis* 1992; **145**: 918–921.
5. Wardlaw AJ, Dunette S, Gleich GJ, Collins JV, Kay AB. Eosinophils and mast cells in bronchoalveolar lavage in subjects with mild asthma. Relationship to bronchial hyperreactivity. *Am Rev Respir Dis* 1988; **137**: 62–69.
6. Robinson DS, Bentley AM, Hartnell A, Kay AB, Durham SR. Activated memory T helper cells in bronchoalveolar lavage fluid from patients with atopic asthma: relation to asthma symptoms, lung function, and bronchial responsiveness. *Thorax* 1993; **48**: 26–32.
7. Smith DL, Deshazo RD. Bronchoalveolar lavage in asthma. An update and perspective. *Am Rev Respir Dis* 1993; **148**: 523–532.
8. Arm JP, Lee TH. The pathobiology of bronchial asthma. *Adv Immunobiol* 1992; **51**: 323–382.
9. Unanue ER, Allen PM. The basis for the immunoregulatory role of macrophages and other accessory cells. *Science* 1987; **236**: 551–557.
10. Sibille Y, Reynolds HY. Macrophages and polymorphonuclear neutrophils in lung defense and injury. *Am Rev Respir Dis* 1990; **141**: 471–501.
11. Holt PG, Oliver J, Bilyk N, *et al.* Downregulation of the antigen presenting cells function(s) of pulmonary dendritic cells *in vivo* by resident alveolar macrophages. *J Exp Med* 1993; **177**: 397–407.
12. Poulter LW, Janossy G, Power C, Screenan S, Burke C. Immunological/ physiological relationship in asthma: potential regulation by lung macrophages. *Immunol Today* 1994; **15**: 258–261.
13. Kelly C, Ward C, Stenton CS, Bird G, Hendrick DJ, Walters EH. Number and activity of inflammatory cells in bronchoalveolar lavage fluid in asthma and their relation to airway hyperresponsiveness. *Thorax* 1988; **43**: 684–692.
14. Rankin JA. The contribution of alveolar macrophages to hyperreactive airway disease. *J Allergy Clin Immunol* 1989; **83**: 722–729.
15. Chanez P, Vignola AM, Lacoste P, Michel FB, Godard P, Bousquet J. Increased expression of adhesion molecules (ICAM-1 and LFA-1) on alveolar macrophages from asthmatic patients. *Allergy* 1993; **48**: 576–580.
16. Robinson DS, Ying S, AM Bentley, *et al.* Relationship among numbers of bronchoalveolar lavage cells expressing messenger ribonucleic acid for cytokines, asthma symptoms, and airway methacholine responsiveness in atopic asthma. *J Allergy Clin Immunol* 1993; **92**: 397–403.
17. Williams J, Johnson S, Mascali JJ, Smith H, Rosenwasser LJ, Borish L. Regulation of low-affinity IgE receptor (CD23) expression on mononuclear phagocytes in normal and asthmatic subjects. *J Immunol* 1992; **149**: 2823–2829.
18. Barnes PJ. A new approach to the treatment of asthma. *New Engl J Med* 1989; **321**: 1517–1527.
19. Barnes PJ, Pederson S. Efficacy and safety of inhaled corticosteroids in asthma. *Am Rev Respir Dis* 1993; **148**: S1–S26.
20. Van Hal PThW, Hoogsteden HC. Inflammatory mediators and glucocorticoids. In: Bray MA, Anderson WH, eds. *Lung Biology in Health and Disease. Mediators of pulmonary inflammation.* New York: M Dekker, 1991; **54**: 593–617.
21. De Waal RMW. The anti-inflammatory activity of glucocorticoids. *Mol Biol Rep* 1994; **19**: 81–88.
22. Schleimer RP. Effects of glucocorticoids on inflammatory cells relevant to their therapeutic applications in asthma. *Am Rev Respir Dis* 1990; **141**: S59–S69.
23. Goulding NJ, Guyre PM. Glucocorticoids, lipocortins and the immune response. *Curr Opin Immunol* 1993; **5**: 108–113.
24. Laitinen LA, Laitinen A, Haahtela T. A comparative study of the effects of an inhaled corticosteroid, budenoside, and of a $\beta_2$-agonist, terbutaline, on airway inflammation in newly diagnosed asthma: a randomized, double-blind, parallel-group controlled trial. *J Allergy Clin Immunol* 1992; **90**: 32–42.
25. Jeffery PK, Godfrey RW, Ädelroth E, Nelson F, Rogers A, Johansson S-Ä. Effects of treatment on airway inflammation and thickening of basement membrane reticular collagen in asthma: a quantitative light and electron microscopy study. *Am Rev Respir Dis* 1992; **45**: 890–899.
26. Trigg CJ, Manolitsas ND, Wang J, *et al.* Placebo-controlled immunopathologic study of four months of inhaled corticosteroids in asthma. *Am J Respir Crit Care Med* 1994; **150**: 17–22.
27. Wilson JW, Djukanovic R, Howarth PH, Holgate ST. Inhaled beclomethasone dipropionate downregulates airway lymphocyte activation in atopic asthma. *Am J Respir Crit Care Med* 1994; **149**: 86–90.
28. Barnes PJ. Inhaled glucocorticosteroids for asthma. *New Engl J Med* 1995; **332**: 868–875.
29. Corrigan CJ, Kay AB. CD4 T-lymphocyte activation in acute severe asthma: relationship to disease severity and atopic status. *Am Rev Respir Dis* 1990; **141**: 970–977.
30. Walker C, Virchow Jr J-C, Bruijnzeel PLB, Blaser K. T cell subsets and their soluble products regulate eosinophilia in allergic and nonallergic asthma. *J Immunol* 1991; **146**: 1829–1835.
31. Kerstjens HAM, Brand PLP, Hughes MD, *et al.* A comparison of bronchodilator therapy with or without inhaled corticosteroid therapy for obstructive airway disease. *New Engl J Med* 1992; **327**: 1413–1419.
32. Brand PLP, Kerstjens HAM, Postma DS, *et al.* Long-term multicentre trial in chronic nonspecific lung disease: methodology and baseline assessment in adult patients. *Eur Respir J* 1992; **5**: 21–31.
33. ATS Task Group. Standards for the diagnosis and care of patients with chronic obstructive pulmonary disease and asthma. *Am Rev Respir Dis* 1987; **136**: 225–231.
34. Cockroft DW, Kilian DN, Mellon JJA, Hargreave FE. Bronchial hyperreactivity to inhaled histamine: a method and clinical survey. *Clin Allergy* 1977; **7**: 235–243.
35. Montefort S, Holgate ST. Adhesion molecules and their role in inflammation. *Resp Med* 1991; **85**: 91–99.
36. Kenny AH, O'Hare MJ, Gusterson BA. Cell-surface peptidases as modulation of growth and differentiation. *Lancet* 1989; **2**: 785–787.
37. Van Hal PThW, Hopstaken-Broos JPM, Prins A, *et al.* Potential anti-inflammatory effects of IL-4: stimulation of human monocytes, macrophages, and endothelial cells by IL-4 increases aminopeptidase-N activity (CD13; EC 3.4.11.2). *J Immunol* 1994; **153**: 2718–2728.
38. Ziegler-Heitbrock HWL, Ulevitch RJ. CD14: cell surface receptor and differentiation marker. *Immunol Today* 1993; **14**: 121–125.
39. Van de Winkel JGJ, Anderson CL. Biology of human immunoglobulin G Fc receptors. *J Leukocyte Biol* 1991; **49**: 511–524.
40. Joseph M, Tonnel A-B, Torpier G, Capron A, Arnoux B, Benveniste J. Involvement of immunoglobulin E in the secretory processes of alveolar macrophages from asthmatic patients. *J Clin Invest* 1983; **71**: 221–230.
41. Van Hal PThW, Hoogsteden HC, Te Velde AA, Wijkhuijs JM, Figdor CG, Hilvering C. IL-4-induced maturation of monocytes/macrophages is inhibited by glucocorticoids. Submitted.
42. Hench PS, Kendall EC, Slocumb CH, Polley HF. The effect of a hormone of the adrenal cortex (17-hydroxy-11-dehydrocorticosterone: compound E) and of pituitary adrenocorticotropic hormone on rheumatoid arthritis. *Proc Staff Meet, Mayo Clin* 1949; **24**: 181–197.
43. Clark TJH. Effect of beclomethasone dipropionate delivered by aerosol in patients with asthma. *Lancet* 1972; **i**: 1361–1364.
44. Fabbri L, Burge PS, Croonenborgh L, *et al.* Comparison of fluticasone propionate with beclomethasone dipropionate in moderate to severe asthma treated for one year. International study group. *Thorax* 1993; **48**: 817–823.
45. Evans PM, O'Connor BJ, Fuller RW, Barnes PJ, Chung KF. Effect of inhaled corticosteroids on peripheral eosinophil counts and density profiles in asthma. *J Allergy Clin Immunol* 1993; **91**: 643–649.
46. Kuo HP, Yu TR, Yu CT. Hypodense eosinophil number relates to clinical severity, airway hyperresponsiveness and response to inhaled corticosteroids in asthmatic subjects. *Eur Respir J* 1994; **7**: 1452–1459.
47. Springer TA. Adhesion receptors of the immune system. *Nature* 1990; **346**: 425–434.
48. Haskard DO, Lee TH. The role of leukocyte-endothelial interactions in the accumulation of leukocytes in allergic inflammation. *Am Rev Respir Dis* 1992; **145**: S10–S13.
49. Bochner BS, Undem BJ, Lichtenstein LM. Immunological aspects of allergic asthma. *Annu Rev Immunol* 1994; **12**: 295–335.
50. Van Hal PThW, Hopstaken-Broos JPM, Wijkhuijs JM, te Velde AA, Figdor CG, Hoogsteden HC. Regulation of aminopeptidase-N (CD13) and FcRIIb (CD23) expression by IL-4 depends on the stage of maturation of monocytes/macrophages. *J Immunol* 1992; **149**: 1395–1402.
51. Beekhuizen H, Blokland I, Corsel-van Tilburg AJ, Koning F, van Furth R. CD14 contributes to the adherence of human monocytes to cytokine-stimulated endothelial cells. *J Immunol* 1991; **147**: 3761–3767.
52. Rinehart JJ, Wuest D, Ackermann GA. Corticosteroid alteration of human monocyte to macrophage differentiation. *J Immunol* 1982; **129**: 1436–1440.

ACKNOWLEDGEMENTS. We gratefully acknowledge Professor Dr C. Hilvering and Professor Dr R. Benner for their continuous support and advice, and Mrs J. M. J. van Everdingen-Quartel for secretarial assistance. This study was financially supported by Glaxo Wellcome B.V., the Netherlands.

**Received 14 June 1996; accepted in revised form 6 September 1996**
